# Identifying tumor promoting genomic alterations in tumor-associated fibroblasts via retrovirus-insertional mutagenesis

**DOI:** 10.18632/oncotarget.21881

**Published:** 2017-10-16

**Authors:** Lijie Rong, Yangyang Bian, Shubai Liu, Xiaoman Liu, Xiao Li, Haiyang Liu, Jinxue Zhou, Jirun Peng, Henghui Zhang, Hongsong Chen, Zhihai Qin

**Affiliations:** ^1^ Medical Research Center, The First Affiliated Hospital of Zhengzhou University, Zhengzhou University, Zhengzhou, 450052, China; ^2^ Key Laboratory of Protein and Peptide Pharmaceuticals, Chinese Academy of Sciences-University of Tokyo, Beijing, 100101, China; ^3^ Joint Laboratory of Structural Virology and Immunology, Institute of Biophysics, Chinese Academy of Sciences, Beijing, 100101, China; ^4^ University of Chinese Academy of Sciences, Beijing, 100049, China; ^5^ Henan Tumor Hospital, Zhengzhou, 450008, China; ^6^ Department of Surgery, Beijing Shijitan Hospital, Capital Medical University, Beijing, 100038, China; ^7^ Capital Medical University Clinical Cancer Center, Beijing, 100038, China; ^8^ Ninth Clinical Medical College of Peking University, Beijing, 100038, China; ^9^ Peking University People's Hospital, Peking University Hepatology Institute, Beijing, 100044, China

**Keywords:** Tumor-associated fibroblasts, retrovirus-insertional mutagenesis, tumor-promoting gene, co-injection model, Ttl

## Abstract

Tumor-associated fibroblasts (TAFs) are often essential for solid tumor growth. However, few genetic or epigenetic alterations have been found in TAFs during the progression of solid tumors. Employing a tumor-stromal cell co-injection model, we adapted here retroviral-insertional mutagenesis to stromal cells to identify novel tumor-associated genes in TAFs. We successfully identified 20 gene candidates that might modulate tumor growth if altered in TAFs at genomic level. To validate our finding, the function of one of the candidate genes, tubulin tyrosine ligase (*Ttl*), was further studied in TAFs from fibrosarcoma, colon, breast and hepatocarcinoma. We demonstrated that down-regulated *TTL* expression in TAFs indeed promoted tumor growth in mice. Interestingly, decreased expression of *TTL* in tumor stromal cells also correlated with poor outcome in human colon carcinoma. Thus, the co-injection model of tumor cells with retrovirus-modified fibroblasts proved a valid method to identify tumor-modulating genes in TAFs, allowing for a deeper insight into the role of the stroma for tumor development.

## INTRODUCTION

Spontaneous genetic or epigenetic aberrations are hallmarks of tumor cells, but are also discovered in some stromal cells [1, 2]. Heterozygosity is often lost in stromal cells that surround tumor cells in human epithelial tumor tissues [3–6]. Some studies argue for rare genetic aberrations in stromal cells [7] that might originate from tumor cells after epithelial–mesenchymal transition [8]. It is widely acknowledged that co-evolution with tumor cells favors genetically or epigenetically abnormal tumor stromal cells [1, 9]. Most aberrations were found in fibroblast-like stromal cells, also known as tumor-associated fibroblasts (TAFs). Altered genes including *Pten*[10], *Tgfbr2*[11], *Tp53*[3], *Sqstm1*[12], *Ereg*[13], *Ifngr*[14], *Hif1a* and *Vegfa*[15] were identified from genetically engineered mice that limit a technically complicated and time-consuming attempt to only one gene per strain. Using artificial tumor microenvironments, gene expression profiling of fibroblasts co-cultured with tumor cells [16], or growth factor induced TAFs [17], also revealed altered genes in TAFs that functionally associated with tumor growth, such as IDH3α[17]. We here aim for a strategy that systematically identifies multiple functional tumor-associated genes in TAFs from dynamic tumor progression *in vivo*.

Retroviral genomes naturally insert into a cell genome during phylogenesis and ontogenesis [18] where it can activate oncogenes or inactivate tumor-associated genes [19]. The so called retrovirus-insertional mutagenesis has been widely applied to screen tumor-associated genes in tumor cells [19–22]. The tumor-stromal cell co-injection model helped to identify functions of tumor-associated genes in TAFs *in vivo*, for the convenience of genetically manipulating TAFs without altering the tumor cells themselves [23, 24]. Our previous study demonstrated that mouse embryonic fibroblasts (MEFs) upon co-injection mainly differentiated into TAFs [14], making MEFs suitable for this system [14, 20, 23, 25–27]. Combining the advantages of retrovirus-insertional mutagenesis to produce shotgun-mutated MEF libraries and co-injecting them with different tumor cell types into mice, we established a new method to identify multiple functional tumor-associated genes in TAFs. This strategy effectively models complex tumor-stroma interaction *in vivo*. We successfully discovered 20 tumor-associated candidate genes and subsequently confirmed *Ttl*, the gene for tubulin tyrosine ligase as tumor-suppressor gene in TAFs within different mouse tumor models. Strikingly, low *TTL* expression in tissues of human colon cancer or hepatocarcinoma correlated with poor prognosis. These results demonstrate the power of our new strategy to identify relevant tumor-associated genes in TAFs.

## RESULTS

### Establishment of the co-injection model of tumor cells with a fibroblast library containing shotgun gene mutations

We hypothesized that during *in-vivo* tumor progression from co-injected tumor cells and a pool of MEFs mutated by retroviral insertion that we call retro-MEFs, only the subsets of retro-MEFs with tumor-promoting potential survive, proliferate and enrich in the tumor stroma. The retroviral integration sites in the genome DNA of this retro-MEF progeny, further called TA-MEFs and considered equivalent to TAFs, can be identified and will provide the information on tumor-associated genes within the tumor stroma. We first tested the feasibility of all steps of our strategy.

**Figure 1:**
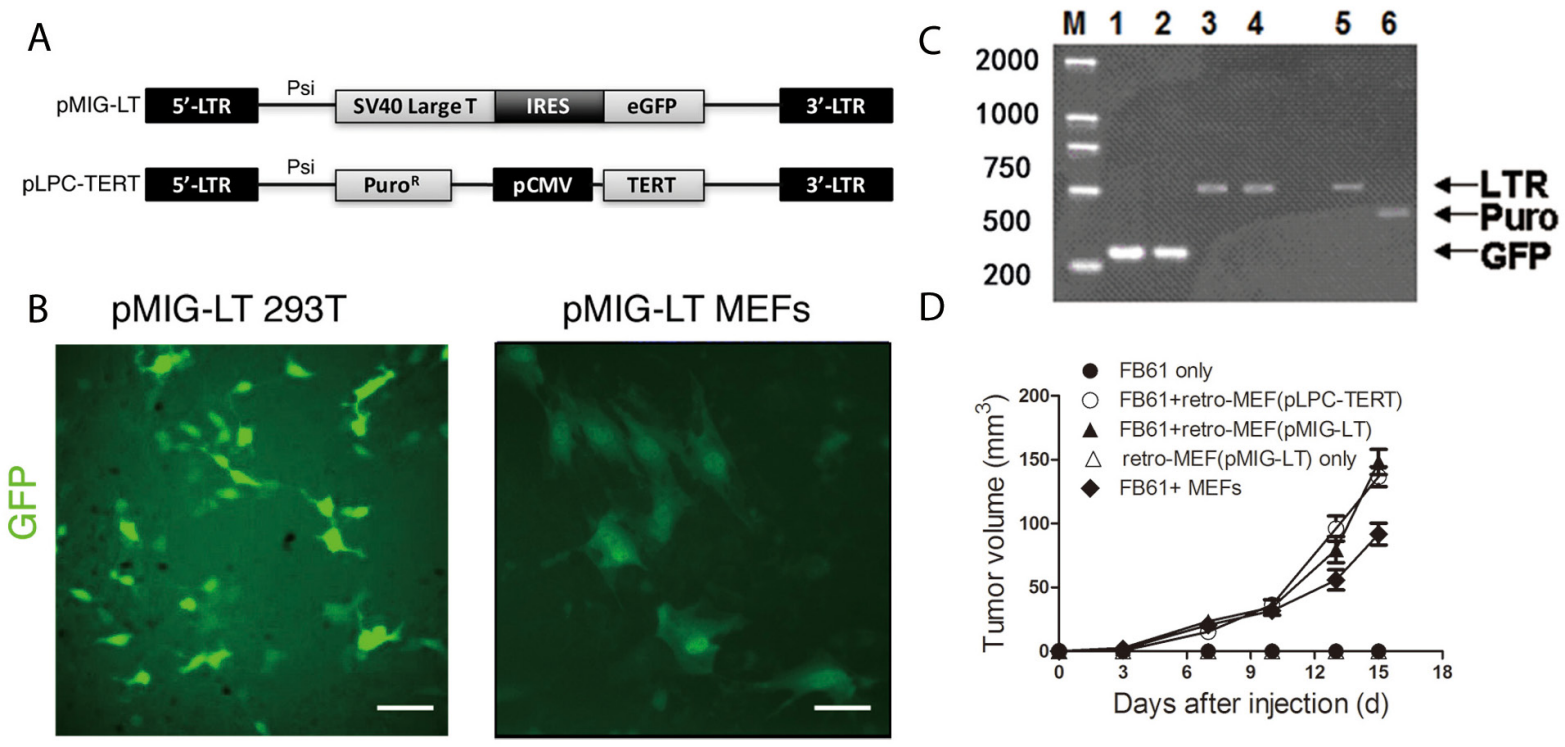
Production of a fibroblast library with shotgun gene mutations (retro-MEFs) **(A)** Schema of the retroviral insertion sequences from the plasmids pMIG-LT and pLPC-TERT. **(B)** 293T cells (left panel) and freshly prepared MEFs (right panel) were transfected with pMIG-LT and cultured on slide for 24 h. Green fluorescence was assessed by fluorescence microscopy. Scale bars, 50 μm; data are representative of two independent experiments. **(C)** Agarose-gel images showing successful transfection. pMIG-LT was used to transfect 293T cells (lanes 1,3) or MEFs (lane 2,4); pLPC-TERT for MEFs (lanes 5,6). Inserted retroviral sequences were assessed by RT-PCR specific for GFP (amplicon size: 278 bp; lanes 1-2), retroviral LTR (amplicon size: 512 bp; lanes 3,4,5) or the puromycin-resistance gene (amplicon size: 441 bp; lane 6). M, marker. **(D)** Retro-MEFs were prepared by infecting MEFs with pMIG-LT (LT-GFP) or pLPC-TERT (TERT-puro) retrovirus. FB61 tumor cells were injected alone or in combination with retro-MEFs as indicated. Injection of retro-MEFs with pMIG-LT retroviral particles served as control. Tumor volumes were monitored over time as indicated. Mean ± SEM, ^**^P< 0.01, two-way ANOVA, n=4 per group; data are representative of two independent experiments.

To estimate the infection efficiency and ensure ample numbers of retro-MEFs for use in the mouse model, we infected freshly prepared MEFs with retrovirus produced by a packaging cell line 293T that was transfected with the pMIG-LT vector (Figure 1A and 1C). After 72 hours, about 30% of the retro-MEFs were GFP^+^ (Figure 1B) and retrovirus-specific LTR amplicons from MEFs’ genomic DNA demonstrated successful integration (Figure 1C). In our previous study, the proportion of GFP^+^ TAFs in the tumor established by the co-injection of GFP^+^ MEFs with tumor cells became less than 2% after 16 days [14]. So, it was technically challenging to purify these GFP^+^ MEFs. We tested a pLPC-TERT retrovirus containing a puromycin-resistance gene (Figure 1A) and found it successfully integrating into the genome of infected MEFs too (Figure 1C). These MEFs were easily enriched by puromycin selection that effectively eliminated e.g. contaminating tumor cells or host fibroblasts. We next investigated the capacity of retro-MEFs to support tumor development. If co-injected with FB61 fibrosarcoma cells, MEFs infected with pMIG-LT or pLPC-TERT retrovirus, equally supported FB61 tumor growth (Figure 1D). Therefore, the pLPC-TERT retrovirus was used in the following study.

**Figure 2:**
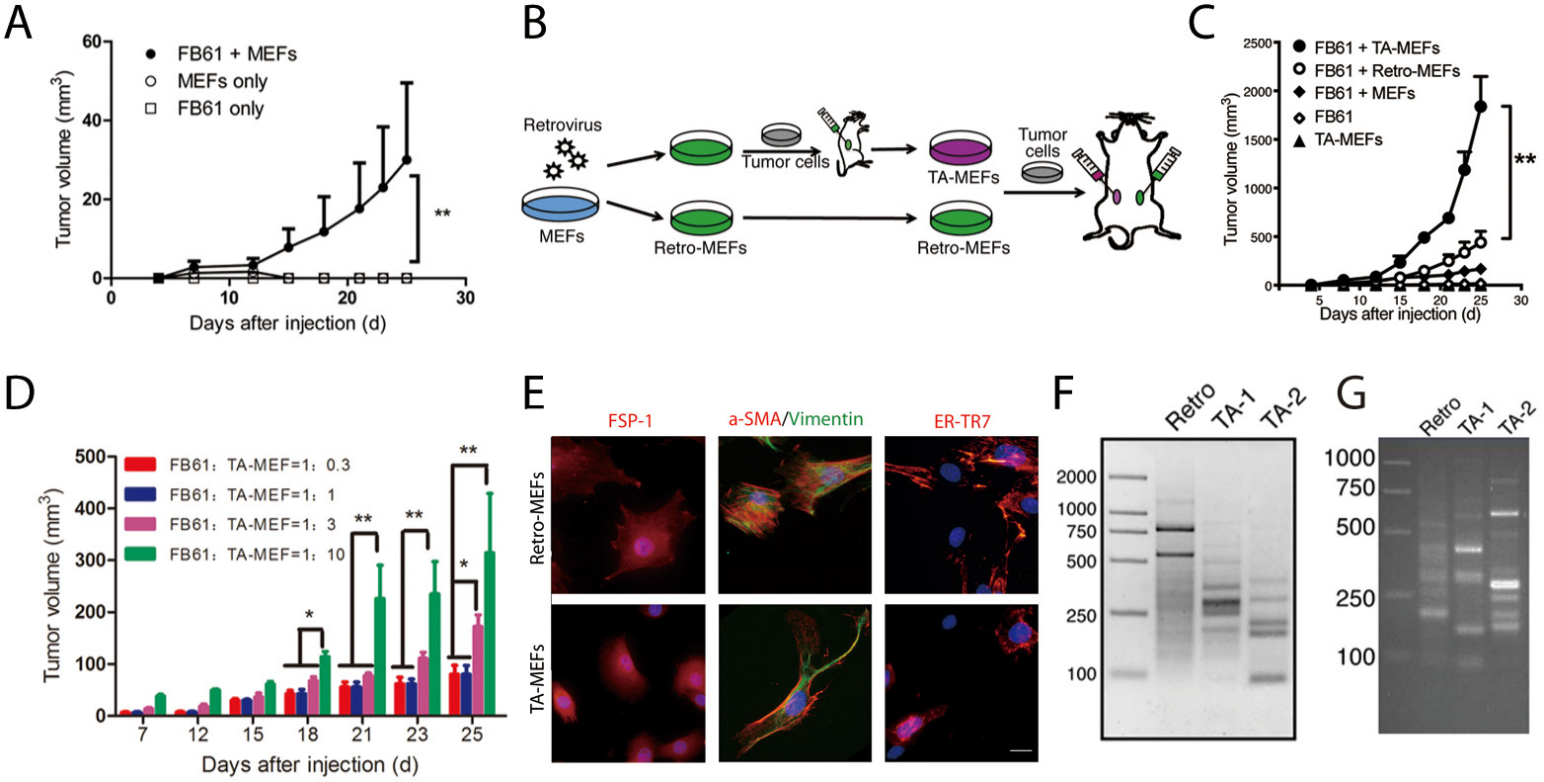
*In vivo* discovery of tumor-associated genes in TA-MEFs **(A)** BALB/c mice were subcutaneously injected with MEFs in combination with FB61 tumor cells. Injection of FB61 tumor cells or MEFs alone served as control. Tumor volumes were monitored over time as indicated. Mean ± SEM, ^**^P< 0.01, two-way ANOVA, n = 6 per group; data are representative of three independent experiments. **(B)** Schema for the functional development of TA-MEFs from shotgun transduced MEFs (retro-MEFs) via primary tumor passage. **(C)** BALB/c mice were subcutaneously injected with MEFs, retro-MEFs or TA-MEFs in combination with FB61 tumor cells. Injection of FB61 tumor cells or larger number of TA-MEFs (5×10^6^) alone served as control. Tumor volumes were monitored over time as indicated. Mean ± SEM, ^**^P< 0.01, two-way ANOVA, n=6 per group; data are representative of three independent experiments. **(D)** FB61 cells were mixed with TA-MEFs at different ratios as indicated before co-injection into BALB/c mice. Tumor volumes were monitored over time as indicated. Mean ± SEM, ^*^P< 0.05, ^**^P< 0.01, two-way ANOVA, n=5 per group; data are representative of two independent experiments. **(E)** TA-MEFs isolated from tumors established by co-injection of retro-MEFs and FB61 tumor cells were stained for FSP-1, α-SMA/vimentin or ER-TR7 and assessed by confocal microscopy. Nuclei were counterstained with DAPI. Scale bars, 20 μm; data are representative of two independent experiments. **(F, G)** LAM-PCR was performed for retro-MEFs and TA-MEFs isolated from FB61 (F) and TS/A (G) tumors. TA-1 and TA-2 were two different batches of TA-MEFs isolated from two different tumors. Data are representative of three independent experiments.

Addressing cell-dose dependencies and specificity in the co-injection model, we started with FB61 cells in combination with non-mutated MEFs. Tumors were successfully established after subcutaneous injection of MEFs (1×10^5^) combined with low numbers of FB61 cells (2×10^4^), while MEFs or FB61 cells alone did not establish tumors (Figure 2A). We further co-injected FB61 cells with parental retro-MEFs or TA-MEFs, and injected very large numbers of TA-MEFs (5×10^6^) alone to test their tumorigenicity (Figure 2B). Tumor-derived TA-MEFs were not only superior to retro-MEFs or normal MEFs in supporting tumor development (Figure 2C), but dose-dependently augmented tumor growth (Figure 2D). During TA-MEF purification from tumor tissues, the efficiency of puromycin selection was routinely assessed by parallel *in-vitro* culture of FB61 tumor cells in the presence of puromycin (see Supplementary Figure 1). To ensure absent tumor cell contamination in the TA-MEF preparations, the selective pressure of puromycin was kept in the TA-MEFs cultures for an additional week. Final TA-MEFs preparations did not contain cells with the hematopoietic or endothelial cell markers CD45 and CD31 (not shown). All cells expressed the mesenchymal cell markers vimentin, α-smooth muscle actin (SMA), fibroblast-specific protein (FSP)-1 and ER-TR7 (Figure 2E). The staining result is consistent with our previous finding that the progeny of MEFs in such co-injection models mostly displayed an appearance of fibroblasts. Our experimental setup did not exclude that a small proportion of co-injected MEFs might have differentiated into other cell types [14]. Since these were not tumor cells, we assumed them negligible for the overall effects. The products of LAM-PCR from retro-MEFs out of FB61 and TS/A tumor tissues appear as continuum of amplicon length, whereas a decreased diversity of retroviral integration sites in the TA-MEFs is represented by defined bands with very high intensity against a reduced background of amplicon length (Figure 2F-2G).

Taken together, TA-MEFs were able to support substantial tumor growth with 2×10^4^ co-injected tumor cells, an amount that was used in all following experiments. Fulfilling a prerequisite condition for our screening strategy, selective enrichment of limited numbers of LTR-containing amplicons during tumor progression indicated that the TA-MEFs derived from retro-MEFs indeed functioned as TAFs.

### Identification of retrovirus integration sites

Twelve batches of TA-MEFs re-isolated from co-injection models with FB61 (6/12), TS/A (4/12) and MCA-205 (2/12) were subjected to LAM-PCR. For each batch, the three most abundant bands were cloned and sequenced. We obtained sequence data from 68/144 colonies from the FB61-tumor model whereas 55 of them contained LAM-PCR adaptor and retroviral LTR sequences. DNA sequences between LAM adaptor and retroviral LTR were compared to mouse genomic DNA sequences, 16/55 showed similarities higher than 95%. These numbers suggested valid amplification of TAF genomic DNA adjacent to the insertion site and successful identification of retrovirus insertion sites. Increasing the confidence in the identified integration sites, only sites located within, or up/down-stream of a gene within a window size of 30 kb were considered further. Finally, the FB61 model revealed 7 different insertion sites while an altered *Ttl* gene was found twice. Using the same screening strategy, we identified 5 and 2 additional insertion sites from the TS/A and MCA-205 model, respectively.

Totally, our co-injection strategy identified 20 tumor-associated candidate genes from TAFs that located in 14 retroviral insertion sites (Table 1). We reanalyzed four published GSE datasets (GEO: GSE40595, GSE38666, GSE45001, GSE26910) [28–32] from the NCBI GEO database that focused on gene expression differences between tumor stroma and normal stroma, using the online shinyGEO tool [33] (http://bioinformatics.easternct.edu/shinyGEO/). Underlining the high relevance of our identified gene candidates for tumor development, 16/20 genes including *Ttl* were found significantly altered (Table 2, detailed information in Supplementary Table 1).

**Table 1:** Summary of retroviral insertion sites in the genome and tumor-associated candidate genes in TAFs from three mouse tumor models

Tumor model	Insertion ID	Symbol	Subclones identified	Retroviral insertion	Summary of function
FB-61	FB-1	*Ttl*	2	Exon 4	Post-translational tyrosination of the detyrosinated α-tubulin
	FB-2	*Hist1h4m*	4	Exon 1	Transcription regulation
	FB-3	*Coro2b*	2	Intron 7	Binds to F-actin and to vinculin
	FB-4	*Slc43a2*	1	Exon 7	Large neutral amino acids transportation
	FB-5	*Fcho2*	2	Intron 1	Imposing and stabilizing particular membrane curvatures
	FB-6	*Cct3*	4	19.9 kb up	Chaperonin
	FB-6	*Rhbg*	4	22.8 kb up	Ammonium transporter
	FB-7	*Zfx*	1	28.9 kb up	Potential transcriptional activator
MCA-205	M-1	*Bmx*	2	11.8 kb up	Intracellular signaling cascade
	M-1	*Pir*	2	Intron 1	NFI/CTF1 cofactor
	M-2	*Tex13*	2	24.2 kb up	Unknown
TS/A	TS-1	*Stat3*	1	0.5 kb up	STAT transcription factor
	TS-2	*Stab1*	5	Exon 6	Scavenger receptor
	TS-3	*Abt1*	2	8.2 kb up	Transcription co-activator
	TS-3	*Btn1a1*	2	29.2 kb down	Functions in the secretion of milk-fat droplets
	TS-4	*S100a2*	4	2.0 kb down	Ca2+-binding S100 protein
	TS-4	*S100a3*	4	7.9 kb up	Ca2+-binding S100 protein
	TS-4	*S100a4*	4	11.5 kb up	Ca2+-binding S100 protein(Fibroblast specific protein)
	TS-5	*Hnrpl*	2	21.2 kb up	Nucleic acid and nucleotide binding
	TS-5	*Nfkbib*	2	22.2 kb up	NF-kappa B inhibitor

**Table 2:** Appearance of tumor-associated candidate genes from TAFs identified in this study in published datasets comparing tumor stroma (TAF) and normal stroma (NF) from human tissues

Symbol	GSE accessions	FC = TAF/NF^*^	p-value	Tissue type
*PIR*	GSE26910	0.45	0.003	Breast
*S100A4*	GSE26910	0.33	0.002	Prostate
*SLC43A2*	GSE38666	4.00	0.009	Ovary
*S100A2*	GSE38666	20.00	<0.001	Ovary
*STAB1*	GSE38666	9.09	<0.001	Ovary
*S100A4*	GSE38666	8.33	0.001	Ovary
*TTL*	GSE40595	0.26	<0.001	Ovary
*S100A4*	GSE40595	9.55	<0.001	Ovary
*CCT3*	GSE40595	9.61	<0.001	Ovary
*FCHO2*	GSE40595	2.49	0.003	Ovary
*STAT3*	GSE40595	5.43	<0.001	Ovary
*S100A2*	GSE40595	3.94	<0.001	Ovary
*STAB1*	GSE40595	4.47	<0.001	Ovary
*BMX*	GSE45001	0.15	<0.001	Bile duct
*S100A3*	GSE45001	9.34	<0.001	Bile duct
*S100A2*	GSE45001	4.88	0.003	Bile duct

^*^Genes with fold change (FC) values >2.0 or <0.5 and p <0.01 were considered significantly up- or down-regulated.

The location of retrovirus integration suggests the direction of regulation of the candidate genes. Four gene candidates (*Ttl, Hist1h4m, Slc43a2, Stab1*) inserted in an exon with putative loss-of-function. Asking for the biological relevance of our findings in TAFs, we focused the following study on the functional role of *Ttl* that encodes an enzyme catalyzing posttranslational tyrosination of α-tubulin [34]. Lafanechere *et al.* showed a tumor-suppressor role for *Ttl* in NIH-3T3 fibroblasts [35], but considered these cell solely as tumor cells. Their study does not reflect on *Ttl* as a tumor-suppressor gene from the non-tumorous cell compartment within the tumor microenvironment as we specifically do here [14].

### *Ttl* is a tumor suppressor gene in stromal cells during tumor progression

**Figure 3:**
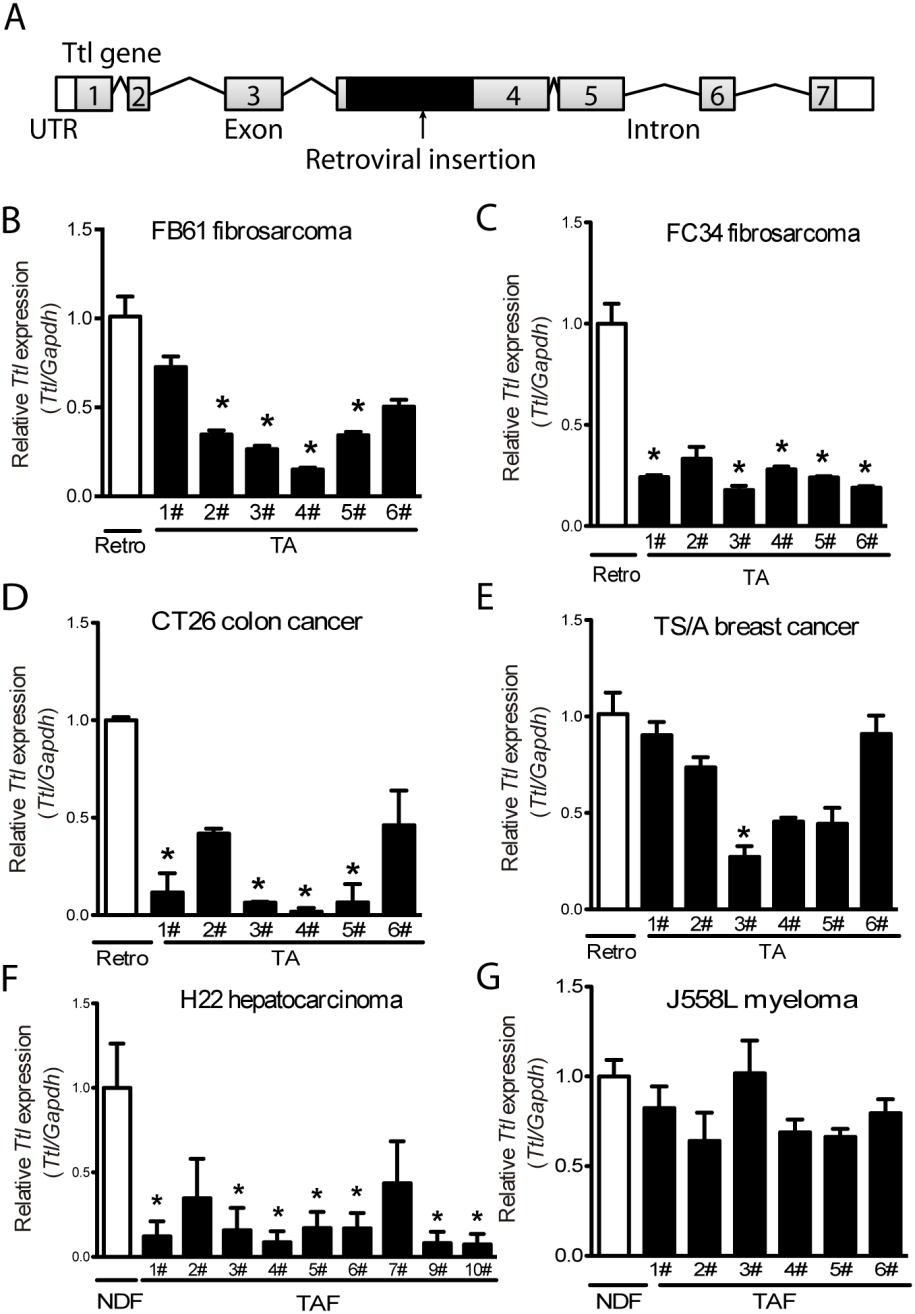
Suppressed stromal *Ttl* expression in different mouse tumor models **(A)** Schema of the retroviral integration site in the *Ttl* gene. Grey boxes with numbers represent the exons. Lines represent introns. Black boxes indicate the inserted retroviral genome. White boxes indicate untranslated regions (UTR). **(B-E)** *Ttl* mRNA levels in retro-MEFs and TA-MEFs (TA) isolated from (B) FB61, (C) FC34, (D) CT26, or (E) TS/A tumors. **(F-G)** *Ttl* mRNA levels in normal dermal fibroblasts (NDFs) and tumor-associated fibroblasts (TAFs) isolated from the subcutaneously transplanted (F) H22, n=10, and (G) J558L tumors, n=6. (B-G) Each bar indicates RT-PCR replicates of one batch of stromal cells re-isolated from a distinct tumor. Mean ± SEM, ^*^P< 0.05, Student’s *t* test, n=6 per model; data are representative of two independent experiments.

We next studied the tumor-suppressing capacity of *Ttl* in TAFs. The retrovirus insertion site within the exon 4 of *Ttl* indicated suppression of the *Ttl* expression (Figure 3A). Indeed, *Ttl* mRNA levels of TA-MEFs from the FB61 model were significantly lower compared with parental retro-MEFs (Figure 3B) - a finding repeated in TA-MEFs from most FC34, CT26 tumors and one TS/A tumor (Figure 3C-3E). To exclude extraneous effects of fibroblasts, host TAFs from H22 and J558L tumors without MEFs co-injection were isolated. In H22 TAFs, *Ttl*-mRNA levels were lower than that in normal dermal fibroblasts (Figure 3F). Notably, *Ttl* was not suppressed in the majority of the TA-MEFs from the TS/A tumors (Figure 3E) or J558L host TAFs (Figure 3G).

**Figure 4:**
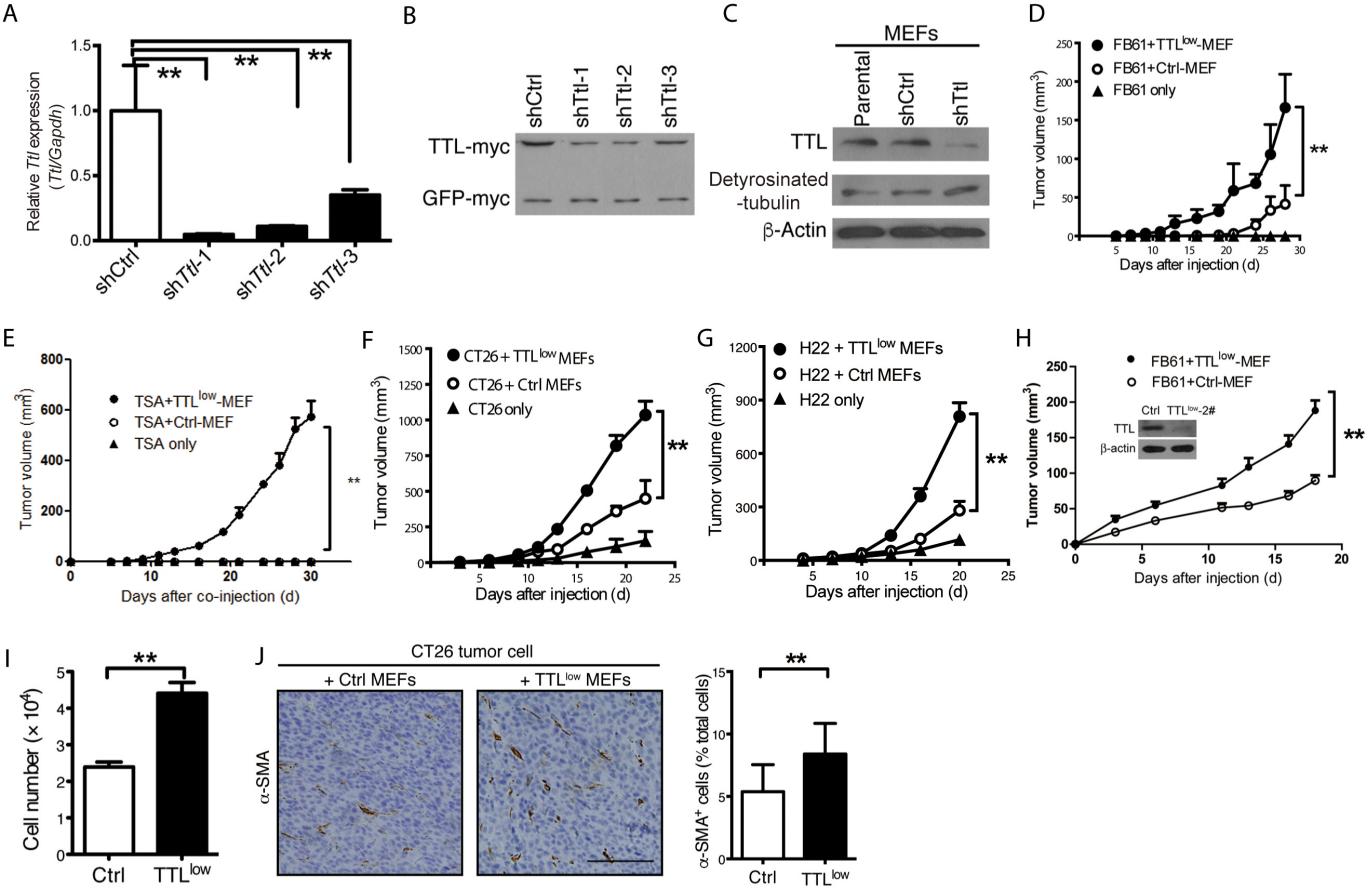
Promoted tumor growth by suppressed stromal *Ttl* expression **(A-C)** Knock-down of *Ttl* in MEFs by three sh*Ttl* sequences. Three sh*Ttl* sequences were cloned into pSUPER.retro.puro plasmid. The 293T cells were transfected with either sh*Ttl* or non-targeting shCtrl vectors, together with a plasmid that carries murine *Ttl* and *GFP* cDNA with independent myc-tags for the expression of murine *Ttl* in the 293T cells, which is human origin. The efficacy of sh*Ttl* was determined after 24 hours. (A) Real time RT-PCR. Mean ± SEM, ^**^P< 0.01, Student’s *t* test, n=3 RT-PCR replicates; data are representative of two independent experiments. (B) Western-blot analysis using an antibody specific for the myc-tag to show TTL-myc (∼45 kDa) in comparison to GFP-myc (∼27 kDa) as control for transfection efficiency. Data are representative of two independent experiments. (C) MEFs were transduced with sh*Ttl*-1 (sh*Ttl*) or shCtrl retroviral particles. Western-blot analysis using antibodies specific to TTL (∼45 kDa) and detyrosinated α-tubulin (detyrosinated-tubulin; ∼50 kDa). β-actin (∼ 42 kDa) served as loading control. Data are representative of three independent experiments. **(D-G)** TTL^low^ MEFs (transduced with sh*Ttl*-1) or Ctrl MEFs were co-injected with 2×10^4^ (D) FB61, (E) TS/A, (F) CT26, or (G) H22 tumor cells. As a control, 2×10^4^ tumor cells were injected alone. Tumor volumes were monitored over time as indicated. Mean ± SEM, ^**^P< 0.01, two-way ANOVA, n = 6 per group; data are representative of two of three independent experiments. **(H)** 1×10^6^ TTL^low^ MEFs (transduced with sh*Ttl*-2) or control MEFs were co-injected with 2×10^5^ FB61 tumor cells. Tumor volumes were monitored over time. Mean ± SEM, ^*^P < 0.05, two-way ANOVA, n=3; data are representative of two independent experiments. Insert: Western-blot analysis for *Ttl* and β-actin in MEFs transduced with sh*Ttl*-2 as described for Figure 4C. **(I)** Aormazan-based assay were used for cell proliferation analysis of TTL^low^ MEFs or control MEFs. Mean ± SEM, ^**^P < 0.01, Student’s t-test, n = 6 culture replicates; data are representative of three independent experiments. **(J)** CT26 tumor cells were co-injected into mice with either TTL^low^ or control MEFs. Tumor sections were stained with α-SMA (brown). (Left) Representative images for the staining are shown. Scale bar, 50 μm. (Right) The proportion of α-SMA^+^ cells in the total cells was calculated (counted with nuclear numbers) from 5-6 visual fields (35 visual fields in total) of 3 sections of each tumor. Mean ± SEM, ^**^P < 0.01, Student’s *t*-test, n=6 tumors per group; data are representative of three independent experiments.

To investigate whether TAFs with reduced *Ttl* expression favor tumor growth, we suppressed *Ttl* expression exclusively in MEFs by anti-*Ttl* shRNA (TTL^low^ MEFs) before co-injection (Figure 4A-4C). Very low doses of TTL^low^ MEFs generated by sh*Ttl-1* significantly promoted the *in vivo* growth of FB61, TS/A, CT26 and H22 tumors (Figure 4D-4G). A similar phenomenon was observed if ten times larger numbers of FB61 tumor cells were co-injected at the same ratio with TTL^low^ MEFs transduced with sh*Ttl*-2. We concluded that the effect on tumor growth of TTL^low^ MEFs neither depended on the inactivating *Ttl* sequence nor on the tumor cell number (Figure 4H). We hypothesized that tumor-promoting gene alterations increase proliferation of TAFs enabling them to enrich in a tumor. Indeed, TTL^low^ MEFs proliferated stronger than control MEFs *in vitro* (Figure 4I) and the α-SMA^+^ proportion *in vivo* in CT26 tumors containing TTL^low^ MEFs was moderately increased (Figure 4J). Taken together, these results pointed to tumor-type dependent *Ttl* suppression in TAFs that might be sufficient to promote the growth of certain tumor types independent of TAF proliferation.

### Low *TTL* expression correlates with poor prognosis of human tumors

**Figure 5:**
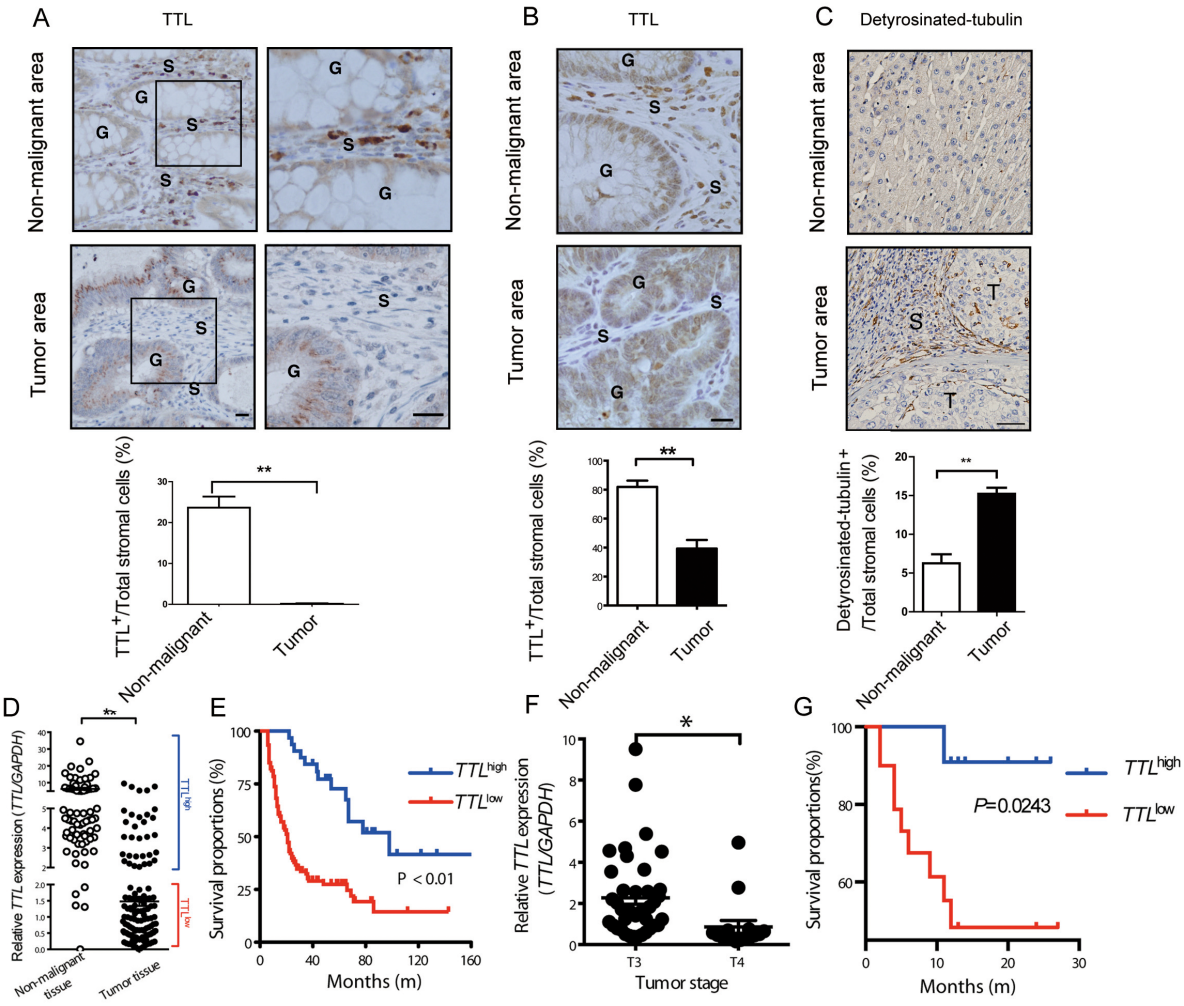
Suppressed TTL expression in stromal cells of human colon and liver cancer tissues **(A)** Paraffin-embedded tissue samples ± 1 cm from the boundary of a tumor nodule (tumor area) or more than 2 cm away from tumor nodules (non-malignant areas) of human colon carcinoma were stained by immunohistochemistry for TTL (brown). (Upper left panel) Representative images from colon areas of one patient; boxed areas enlarged next to original (upper right panel); G, colonic glands; S, stroma; scale bars, 20 μm. **(B)** Representative images of TTL^+^ cells (brown) stained in non-malignant and adenoma areas of the colon tissues from a mouse model of intestinal inflammation-induced carcinogenesis (AOM/DSS). G, colonic glands; S, stromal surrounding the colonic glands; scale bars, 20 μm. **(C)** Tumor and non-tumor areas as described in (A) from human hepatocarcinoma tissues were stained for detyrosinated-tubulin (abbreviated as “Glu-tublin”, brown). Representative images from liver tissue areas from one patient. T, tumor nests; S, stroma. Bars, 50 μm. (A-C, lower panels) Proportions of TTL^+^ or detyrosinated-tubulin^+^ stromal cells were calculated by counting TTL^+^ or detyrosinated-tubulin^+^ stromal cells and all nuclei within the stroma from 35 visual fields (AOM/DSS induced), or 6 visual fields (human colon or liver) tissue sample. Mean ± SEM, ^**^P< 0.01, Student’s *t* test, n=6 tumors per group. **(D)** Human *TTL* mRNA levels in non-malignant colon tissues (n = 72) and colon tumor tissues (n = 77). Scatter plot and mean, ^**^P < 0.0001, Student’s *t* test. Cut-off value for definition of TTL^high^ samples set as 2.0. **(E)** TTL^low^ and TTL^high^ expression in colon tumor tissue was correlated to the overall survival of the colon tumor patients after surgery. *P* < 0.01, log-rank test, n = 72. **(F)** The *TTL* expression levels in colon tumor tissues from T3 (n = 42) and T4 stage (n = 16) were compared. Scatter plot and mean, ^*^P= 0.012, Student’s *t* test. **(G)** TTL^low^ and TTL^high^ expression in liver tumor tissues was defined as described in (D) and correlated to the overall survival times of the hepatocarcinoma patients after surgery. *P*= 0.0243, log-rank test, n = 31.

To investigate whether TTL is relevant in human tumors besides neuroblastoma [36], we examined TTL expression levels in human colon carcinoma and hepatocarcinoma tissues. In colon, TTL was highly expressed in the non-glandular stromal cells of non-malignant areas, but drastically decreased in the TAF-like stromal cells within tumor areas (Figure 5A). A similar phenotype was observed in inflammation induced mouse colon tumors (AOM/DSS, Figure 5B). Detyrosinated α-tubulin was accumulated in stromal cells of human hepatocarcinoma tissues, indicating a TTL^low^-like phenotype (Figure 5C). Underlining this, *TTL*-mRNA levels in colon tumor tissues were significantly lower than that in non-malignant colon tissues (Figure 5D). To evaluate the clinical significance of *TTL*, patients with colon tumors were grouped into TTL^high^ and TTL^low^ based on the relative *TTL* mRNA-levels within the tumor. Eighty months post-surgery, patients in the TTL^high^ group showed better overall survival compared to the TTL^low^ group (Figure 5E), and multivariate survival analysis showed that such grouping based on *TTL* levels significantly impacted survival of patients (Supplementary Table 2). Although *TTL* levels did not correlate with size or metastasis of colon tumors (Supplementary Table 3), the average *TTL* levels in T4-stage tumor tissues was significantly lower than that in T3 stage (Figure 5F). Similarly, low *TTL* levels were also associated with shorter survival times of hepatocarcinoma patients (Figure 5G). However, due to the limited samples, the multivariate survival analysis here did not show such grouping significantly impacted the survival of patients, just as parameters like age, tumor stage and size as well as the degree of liver fibrosis (Supplementary Table 4). Together, the results indicated that low *TTL* levels in human tumors might be a novel indicator of poor prognosis.

## DISCUSSION

More and more studies demonstrated that tumor stroma, especially TAFs are essential in tumor progression. We here for the first time, applied retrovirus insertional mutagenesis to MEFs (TAF precursors) and used them in an *in vivo* tumor-stroma co-injection model to identify tumor-associated genes in TAFs. This strategy successfully identified 20 putative tumor-associated candidate genes in TAFs; some were previously reported as tumor-associated in TAFs, such as *Stat3*[37], *Stab1*[38], and *Nfkbib*[39]. We deliberately choose *Ttl* to underline the biological relevance of the findings using this method and validated the tumor-promoting ability of *Ttl* in TAFs. Differential expression of other candidate genes like *Pir* and *Bmx* between tumor stroma and normal stroma [28–31], indicated the power of the method to address TAFs functions.

Taking in account that the number of processed retroviral insertion sites was relatively limited, we here used a strategy based on cloned LAM-PCR amplicons [40]. This approach enabled the identification of insertion sites that were most abundant in the TAF population, and, as we assumed, the most likely to be functional in TAFs for tumor growth. Confirming known functional tumor-associated genes in stromal cells such as *Stat3*[37] and the proof that the found altered *Ttl* indeed is another tumor-suppressor gene in TAFs supports the strength of this assumption. Of course, this selective approach has some drawbacks. It misses less-enriched insertions and does not allow statistical analysis among the candidate genes or between different tumor models. These questions can be addressed in further studies by deep sequencing.

So far, abnormal *Ttl* expression in the epithelial component of tumors is much better studied than in stromal cells [35, 36, 41–45]. Germline deletion of *Ttl* gene results in perinatal death of mouse [44]. High frequencies (20%) of *TTL* alterations has been found in prostate adenocarcinoma [46]. *Ttl* loss-of-function occurs in different tumor cells, and correlates with tumor progression [35, 36, 41–45]. Most of these tumor cells are the epithelial component of tumor, and only a few studies made mention of varied *Ttl* expression in the stromal components of a tumor, especially in TAFs [41]. Even if *Ttl* levels assessed at mRNA level from whole tumor tissues acceptably substantiates the prognostic value of TTL expression in human tumor tissues, our study suggests that it is much more accurate to address it at protein level in stromal compartment e.g. by immunohistochemistry for TTL or detyrosinated tubulin.

Lafanechere *et al.* elegantly investigated *Ttl* suppression in NIH-3T3 fibroblasts using these fibroblasts as tumor cells [35], while we explicitly focused on the function of *Ttl* in tumor stromal cells. Knock-down of *Ttl* promoted the proliferation of MEFs in *in-vitro* culture. However, in our new *in vivo* model of co-injecting tumor cells and retro-MEFs, promoted tumor growth was not mainly due to the increased TAF numbers or proliferation. Last but not least, injection of only small numbers of tumor cell (2×10^4^) or MEFs alone did not establish tumor in mice. At these cell doses, the mixture of both tumor cells and MEFs was required. All this is in line with our previous work with normal MEFs where arresting proliferation by gamma-radiation did not affect the tumor-promoting capacity of MEFs *in vivo*, in a similar tumor-stroma co-injection model [14]. Therefore, we concluded that promoting TAF proliferation in general is only a minor factor for tumor growth.

Having demonstrated *Ttl* to be a “driver” gene of the tumor-promoting capacity of TAFs, we cannot fully exclude that our strategy identifies proliferation-related genes without tumor-associated function, so called “passenger” genes. Thus, subsequent functional validation of the candidate gene from TAFs during tumor progression is mandatory to confirm its “driver” gene function, as exemplified by *Ttl* here. Notably, with *Ttl*, *S100a4*, *Zfx*, *Cct3*, *Stat3*, *Stab1* and *Bm*x, 7/20 found candidate genes have been reported as proliferation-related in tumor cells too [27, 35, 47–53]. We hypothesize that tumor-promoting gene alterations supporting TAFs proliferation and segregation within the tumor stroma enabled the enrichment of these TAFs and made them accessible. The current retrovirus-insertional mutagenesis is limited by non-random integration and low efficiency. We anticipate that less biased mutation systems such as the Sleeping Beauty transposon will improve the sensitivity of our model.

## MATERIALS AND METHODS

### Patient samples and ethics

Archived formaldehyde-fixed, paraffin-embedded and frozen tumor tissue were obtained the Peking University People’s Hospital and Henan Tumor Hospital samples. Tumor tissue was defined within these samples by the tumor nodules and areas ± 1 cm from the boundary of a tumor nodule. Non-malignant colon tissue was defined from the same samples as areas more than 2 cm distant from any tumor nodule. Fresh colon tissues from surgery that used for RNA preparation were from 77 colon cancer patients, as well as from 72 age- and sex-matched non-tumor patients of the Henan Tumor Hospital. Fresh surgical liver tissues from 31 hepatocarcinoma cancer patients were obtained from the Peking University People’s Hospital. Protocols concerning clinical sample use were approved by the Ethics Committee of Henan Tumor Hospital or Peking University People’s Hospital. Protocols concerning animal use were approved by the Institution of the Animal Care and Use Committee of Institute of Biophysics, Chinese Academy of Sciences (SYXK2013-36).

### Mouse strains, MEFs and tumor cell lines

BALB/c and C57BL/6 mice were purchased from Vital River (China). Age matched (6-8 weeks) female mice were used in all experiments and randomly allocated to experimental and control groups. MEFs were prepared from E13.5 mouse embryos as described [14]. The following cell lines were used: 293T human embryonic kidney, CT26 undifferentiated colon carcinoma (ATCC, LGC Standards), FB61 and FC34 fibrosarcoma (BALB/c)[54], MCA-205 fibrosarcoma (C57BL/6)[55], TS/A mammary adenocarcinoma [56, 57] and J558L myeloma [56, 57]. H22 mouse hepatocarcinoma cells were kindly provided by Dr. Yingxin Xu (The General Hospital of People’s Liberation Army, Beijing, China). If not noted otherwise, cell lines were of murine origin. Cells were cultured in DMEM (293T, CT26) or RPMI 1640 medium supplemented with 10% newborn calf serum and 100 IU/mL penicillin/streptomycin (all from Gibco).

### Plasmids

The pMIG-LT plasmid contained the a green fluorescent protein (GFP) and SV40 large-T fragment from LoxP-HyTK-SV40Tag [58] subcloned to the pMIG vector [59]. Vectors for expressing myc-tagged murine *Ttl* or GFP based on pcDNA4-myc (Invitrogen) were kindly provided by Dr. Guangxia Gao (Institute of Biophysics, Chinese Academy of Sciences, Beijing, China). Oligonucleotides specific for mouse *Ttl* and non-targeting sequences were cloned into pSUPER.retro.puro (Oligo Engine) to produce sh*Ttl*-retroviral particles.

### Retrovirus production and transfection

The 293T cells were transfected with pMIG-LT or pLPC-TERT (Clontech) (Figure 1A) in combination with the pCL–10A1 packaging vector (Imgenex). MEFs were repeatedly infected with freshly prepared retrovirus-containing cell-culture supernatant [60]. We later refered to these cells as retro-MEFs. The 293T cells were transfected with the retroviral vectors pMIG-LT, pLPC-TERT (Clontech) or pSUPER.retro.puro-based sh*Ttl*/Ctrl in combination with the pCL–10A1 packaging vector (Imgenex) using Lipofectamine 2000 (Invitrogen). After 48 and 72 hours of culture, supernatants were collected, filtered (pore size: 0.45 μm; Millipore) and used immediately. To construct retro-MEFs, retrovirus-containing 293T supernatant was mixed with equal volumes of fresh culture medium, supplemented with 8 μg/mL polybrene (Sigma-Aldrich) and added to 3×10^5^ MEFs in a 6-well plate The plate was spun at 1,500×g and 31 °C for 1 hour. After 6 hours at 37 °C, medium was replaced for fresh culture medium. Increasing the infection efficiency, the whole procedure was repeated three times [60].

To knock-down *Ttl* expression in MEFs or in 293T cells co-transfected to express myc-tagged murine *Ttl* and myc-tagged GFP, 1 mL retrovirus-containing 293T supernatant supplemented with 4 μg/mL polybrene was added to 2×10^4^ MEFs or 293T cells. Cells were incubated at 37 °C for 12 hours before the medium was replaced for fresh culture medium. At 36 hours post infection, cells were collected to determine mouse *Ttl* expression. The following sequences specific for the mouse *Ttl* gene and a non-targeting control sequence were synthesized:

sh*Ttl*-1, 5’-gCATTCAgAAAGAgTACTC-3’;
sh*Ttl*-2, 5’-ggCAACgTTTggATTgCAA-3’;
sh*Ttl*-3: 5’-AgTATAATATCTACCTCTA-3’;
shCtrl: 5’-AAgCTgACCCTgAAg-3’.

### Tumor transplantation

Exponentially growing tumor cells from standard culture were harvested, washed twice with PBS and subcutaneously injected into the abdominal region of the mice. In the co-injection model, mice received 2×10^4^ tumor cells and 1×10^5^ retro-MEFs (passages 3-6). Tumor volumes were measured and calculated by (length × width × width)/2 over time.

### Isolation of tumor stromal cells and fibroblasts

Sixteen days after co-injecting tumor cells and retro-MEFs, tumors were surgically removed, cut into small fragments about 3×3×3 mm and digested with 0.48 U/mL collagenase NB4 (Serva) for 30 min at 37 °C. Single-cell suspensions were cultured in fibroblast medium consisting of DMEM supplemented with 10% newborn calf serum and 3 μg/mL puromycin. After <14 days *in vitro*, genomic DNA was extracted. Retro-MEFs subjected to the same *in-vitro* selection procedure served as controls. J558L and H22 tumor cells (1×10^5^) were injected into mice as described above. Twenty days later, cells from the tumors were isolated, cultured in fibroblast medium before suspension-tumor cells were removed by washing the cell layers with fresh fibroblast medium the following day. The remaining adherent TAFs were passaged and identified by immunostaining for the fibroblasts markers α-smooth muscle actin (SMA) and ER-TR7. Normal dermal fibroblasts were isolated from mice with the same genetic background as the host mice of TAFs, as described before [61].

### Linear amplification-mediated (LAM)-PCR

LAM-PCR was performed as described earlier [40]. Briefly, genomic DNA from TA-MEFs was digested with the NlaIII restriction enzyme and subsequently amplified using a biotinylated primer LTRI (BT-LTRI, 5’-Bio-gTTCgCTTCTCgCTTCTgTTCgC-3’). Amplification products were purified with streptavidin-coated Dynabeads M-280 (Invitrogen) and ligated to a linker cassette. Non-biotinylated strands denatured by NaOH (100 mM) served as templates for the subsequent nested PCR with the following primers:

LTRII: 5’-CTCAATAAAAgAgCCCACAACCCCT-3’;
LTRIII: 5’-ACTTgTggTCTCgCTgTTCCTTg-3’.

LAM-PCR Products were separated on 2% agarose gels and purified using MiniElute columns (Qiagen). For each sample, 3 most clearly and brightly enriched gel bands were purified and sub-cloned into the pMD19-T vector (Takara). *E. coli* TOP10 (Invitrogen) were transformed with the resulting plasmids. Plasmids were purified and sequenced using the M13 primer sites. Sequences adjacent to retroviral long terminal repeats (LTR) were blasted against the mouse genome (NCBI) [40]. The sequences of cloning primers were as follows:

Forward: 5'-CCggAAgATCTgCCACCATggATAAAgTTTTAAACAgAg-3';
Reverse: 5'-ggATCCggAATTCTTATgTTTCAggTTCAggg-3'.

All oligonucleotides used within this study were purchased from Sangon Biotech (Shanghai, China).

### Real-time RT-PCR

Total RNA from 1×10^6^ murine fibroblasts or from clinical samples was extracted using TRIzol reagent (Invitrogen) and 2 μg RNA was reversely transcribed by M-MLV reverse transcriptase (Promega). Expression levels of *Ttl* were determined in relation to glyceraldehyde-3-phosphate dehydrogenase (*Gapdh*) using the iQTM SYBR Green Supermix on MyiQTM system (Bio-Rad). The specific primers for real-time PCR were:

### Western-blot analysis

Cells were lysed by RIPA solution before proteins were separated and transferred to nitrocellulose membrane (GE Healthcare) as described before [54]. Briefly, cells were lysed by RIPA solution (50 mM Tris-HCl [pH 7.5], 150 mM NaCl, 1.0% Nonidet P-40, 0.5% sodium deoxycholate, 0.1% sodium dodecyl sulfate, 1 mM EDTA) supplemented with 100 μM phenylmethanesulfonyl fluoride, 25 μg/mL aprotinin, 1 mM sodium orthovanadate, and 50 mM NaF [54]. Proteins from cell extracts were separated using homogenous 10% SDS-PAGE and transferred to a nitrocellulose membrane (GE Healthcare) on a semi-dry transfer device (Bio-Rad). Binding of mouse-specific primary antibodies for *TTL* (1:2,000, SAB1103321, Sigma-Aldrich), β-actin (1:8,000, a5441, Sigma-Aldrich), rabbit polyclonal detyrosinated α-tubulin (1:2,000, ab3201, Millipore), and myc tag (1,000 clone 9E10, Thermo) were visualized using peroxidase-conjugated secondary antibodies (Thermo). The following peroxidase-conjugated secondary antibodies were used: goat anti-rabbit (1:15,000, 32460, Thermo) and goat anti-mouse (1:10,000, 31430, Thermo). Membranes were incubated with a chemiluminescent substrate (Thermo) for 5 minutes and exposed to X-ray film (Kodak).

**Table:** 

Mouse	*Ttl*	Sense: 5’-TgTAgACCATgTAgTTgAggT-3’ Anti-sense: 5’-TACgACTCggggAACCATgAg-3’
	*Gapdh*	Sense: 5’-AggTCggTgTgAACggATTTg-3’ Anti-sense: 5’-TgTAgACCATgTAgTTgAggT-3’
Human	*TTL*	Sense: 5’-CAgCTCTTCggCTTTgACTT-3’ Anti-sense: 5’-GCTgTgggCTggATAAAgAg-3’
	*GAPDH*	Sense: 5’-AAgAAggTggTgAAgCAggC-3’ Anti-sense: 5’-TCCACCACCCTgTTgCTgTA-3’

### Proliferation assay

The cell proliferation was determined using the Cell Counting Kit-8 (Sigma-Aldrich) according to the manufacturers’ instructions. In brief, MEFs were plated in a 96-well plate with the density of 2×10^4^ cells/well, and cultured for 68 hours before the addition of CCK-8 solution. To each well, 10 μL CCK-8 solution were added and cells were cultured for additional 4 hours. The absorbance of the water soluble formazan product was measured at a wavelength of 450 nm using a microplate reader (Bio-rad).

### Immunofluorescent staining and immunohistochemistry

Cells (8×10^3^) were seeded on 10×10 mm coverslips and cultured for 12 hours before staining. Primary antibodies included: rat anti-mouse ER-TR7 (ab51824, Abcam); rabbit anti-mouse CD31 (ab28364, Abcam), vimentin (ab45939, Abcam), FSP-1 (ab27957, Abcam) and CD45 (550539, BD Biosciences); and mouse monoclonal antibody α-SMA (ab5694, Abcam). Secondary antibodies were conjugated with Alexa Fluor 555 or Alexa Fluor 488 (Invitrogen) and nuclei counterstained with 4',6-diamidino-2-phenylindole (Sigma-Aldrich). Slides were assessed by confocal microscopy (FV1000) and images analyzed using the FV10-ASW1.7 Viewer software (both Olympus). Paraffin sections of mouse or human tissues were stained with rabbit anti-mouse TTL (SAB1103321, Sigma-Aldrich) or detyrosinated α-tubulin (ab3201, Millipore) using diaminobenzidine histochemistry kits (Invitrogen). Slides were assessed by brightfield microscopy (DP71, Olympus) and images analyzed using the Image-Pro Plus software (Media Cybernetics).

### Statistical analysis

All data are from two to three independent experiments. Statistical analysis was performed using the GraphPad Prism software (GraphPad). P-values <0.05 were considered statistically significant.

## CONCLUSION

For the first time, we successfully established a method to identify tumor-associated genes in TAFs *in vivo* by applying retrovirus insertional mutagenesis to MEFs in a tumor-stroma co-injection model. TA-MEFs re-isolated from tumors after co-injection exhibit augmented tumor-promoting capacity. Exemplified by suppressed *Ttl* expression, we show new evidence supporting the hypothesis that TAFs containing tumor-promoting genetic/epigenetic changes are selected in the tumor microenvironment. Taken together, we provide a proof of concept how insertional mutagenesis can be used as a novel strategy to identify tumor-associated genes in TAFs.

## SUPPLEMENTARY MATERIALS FIGURE AND TABLES



## Abbreviations

α-SMA: α-smooth muscle actin
GAPDH: glyceraldehyde-3-phosphate dehydrogenase
GFP: green-fluorescent protein
LAM-PCR: linear amplification-mediated PCR
LTR: long terminal repeat
MEF: mouse embryonic fibroblast
TAF: tumor-associated fibroblast
TA-MEF: tumor-associated MEF
TERT: telomerase reverse transcriptase
*Ttl*: tubulin tyrosine ligase
