# Supplementary material for: Identifying tumor promoting genomic alterations in tumor-associated fibroblasts via retrovirus-insertional mutagenesis
Source: Oncotarget. 2017 Oct 16;8(57):97231–45. doi: 10.18632/oncotarget.21881 (PMC5722558; doi:10.18632/oncotarget.21881)
Supplement: Supplementary file 1 [file oncotarget-08-97231-s001.pdf]

Identifying tumor promoting genomic alterations in tumor-associated fibroblasts via retrovirus-insertional mutagenesis

SUPPLEMENTARY MATERIALS

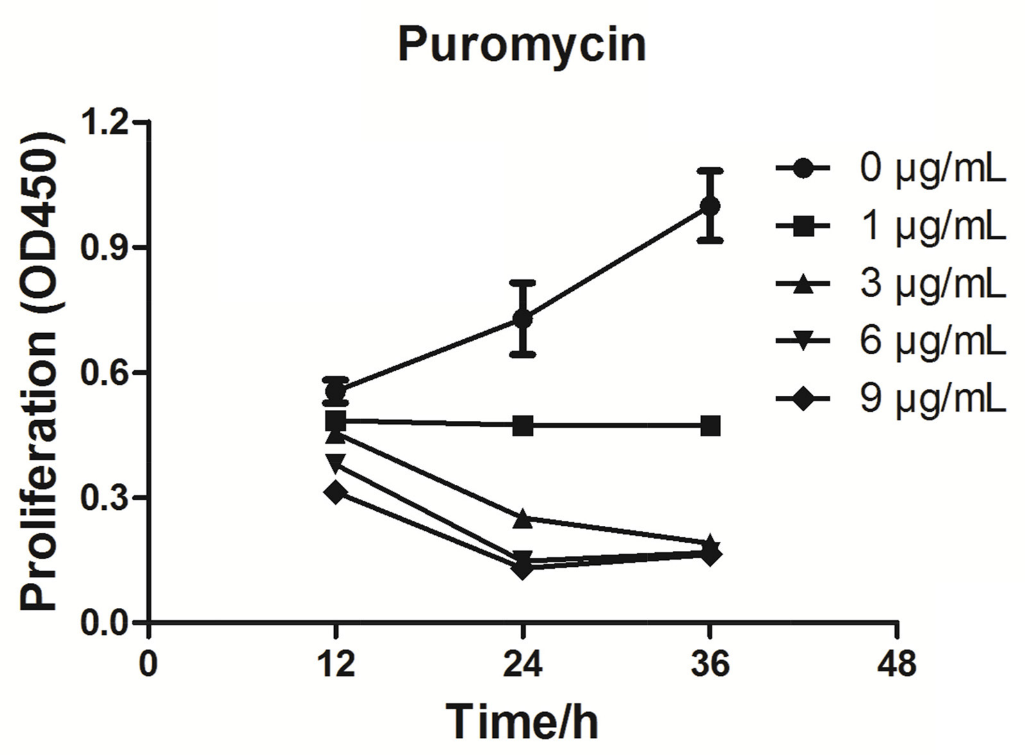

Supplementary Figure 1: Puromycin effectively inhibited proliferation of FB61 cells *in vitro*. FB61 tumor cell line ($5x10^3$ per well) were cultured in a 96-well flat bottom plate in the presence of increasing puromycin concentration indicated. Cell proliferation was assessed by CCK-8 assay. Mean ± SEM of triplicate determination; data are representative of two independent experiments.

Supplementary Table 1: Stromal tissue sources from published data sets used for comparison to tumor-associated candidate genes in TAFs as identified by within this study

ID	Organ	Tumor Type	#samples	
			Tumor stroma	Normal stroma
GSE40595	Ovary	Ovarian cancer	31	8
GSE38666	Ovary	Ovarian cancer	7	8
GSE45001	Bile duct	Intrahepatic cholangiocarcinoma	10	10
GSE26910	Breast	Invasive primary breast cancer	6	6
GSE26910	Prostate	Primary prostate cancer	6	6

Supplementary Table 2: Multivariate survival analysis of the colon tumor patients after surgery (n=77)

Variable	B	SE	Wald	df	Sig.	Exp(B)	95.0% CI for Exp(B)	
							Lower	Upper
Group (by *TTL* mRNA level) *	-2.685	.430	38.944	1	<0.001	.068	.029	.159
sex	-.604	.306	3.901	1	.048	.547	.300	.995
age	-.005	.010	.214	1	.644	.995	.976	1.015
alcohol intake	.620	.350	3.128	1	.077	1.859	.935	3.694
infection	-.235	.354	.439	1	.508	.791	.395	1.583
inflammation	.118	.328	.128	1	.720	1.125	.591	2.140
tumor stage	1.271	.403	9.948	1	.002	3.564	1.618	7.850
tumor diameter	-.021	.039	.301	1	.583	.979	.906	1.057

* The patients were grouped into TTL^{high} and TTL^{low} based on the relative *TTL* mRNA levels within the tumor.

Supplementary Table 3: Correlation of *TTL* mRNA levels in colon tumor tissues from colon cancer patients grouped along patient characteristics

	N	Tumoral *TTL*[*]	*P* value
Total	77		
Sex			
Male	41	1.03 ± 0.16	0.08
Female	36	1.93 ± 0.29	
Ages (yr)			
< 60	39	1.85 ± 0.31	0.75
≥ 60	38	1.72 ± 0.31	
TNM stages			
Tumor diameter			
< 10 mm	31	1.61 ± 0.31	0.83
≥ 10mm	46	1.75 ± 0.42	
Tumor stage			
T2	19	1.34 ± 0.31	0.0001
T3	42	2.28 ± 0.32	
T4	16	0.87 ± 0.31	
Node involvement			
N0	60	1.68 ± 0.24	0.44
N1	17	2.04 ± 0.45	
Metastasis			
M0	52	1.92 ± 0.25	0.21
M1	25	1.23 ± 0.33	
Bowel inflammation			
Yes	28	1.52 ± 0.33	0.36
No	49	1.93 ± 0.28	

[*] Relative human *TTL* expression is shown as *TTL*/*GAPDH*.
[†] Statistics: Tumor stage, one-way ANOVA; others, Student's *t* test.

Supplementary Table 4: Multivariate survival analysis of the liver tumor patients after surgery (n=31)

Variable	B	SE	Wald	df	Sig.	Exp(B)	95.0% CI for Exp(B)	
							Lower	Upper
group*	-.448	.556	.648	1	.421	.639	.215	1.901
sex	.570	.905	.397	1	.528	1.769	.300	10.424
age	-.008	.029	.084	1	.772	.992	.936	1.050
alcohol intake	.649	.583	1.239	1	.266	1.914	.610	6.004
tumor stage	.109	.705	.024	1	.877	1.115	.280	4.437
tumor diameter	.018	.120	.023	1	.880	1.018	.805	1.289
liver fibrosis	.608	1.138	.285	1	.593	1.837	.197	17.094

* The patients were grouped into TTL^{high} and TTL^{low} based on the relative *TTL* mRNA-levels within the tumor.
